# Colonoscopy and polypectomy: beside age, size of polyps main factor for long-term risk of colorectal cancer in a screening population

**DOI:** 10.1007/s00432-021-03532-7

**Published:** 2021-02-04

**Authors:** Kathrin Halfter, Lea Bauerfeind, Anne Schlesinger-Raab, Michael Schmidt, Gabriele Schubert-Fritschle, Dieter Hölzel, Jutta Engel

**Affiliations:** Munich Cancer Registry (MCR), Institute for Medical Information Processing, Biometry and Epidemiology, Ludwig-Maximilians-University (LMU), Marchionini Str. 17, 81377 Munich, Germany

**Keywords:** Colorectal cancer, Cancer registry, Screening, Colonoscopy, Polyps, Adenoma

## Abstract

**Purpose:**

Despite national and international guideline recommendations, few studies have been conducted to estimate the impact of colonoscopy screening on long-term colorectal cancer incidence. Aim of this study was to determine the long-term impact of a full colonoscopy with polypectomy on colorectal cancer incidence in a large screening population.

**Methods:**

In this prospective observational cohort study, a total of 10,947 colonoscopy screening participants from within the scope of the Munich Cancer Registry were consecutively recruited from participating gastroenterology practices and their subsequent colorectal cancer incidence assessed. Predictive factors associated with colorectal cancer were also evaluated in univariate and multivariate analyses.

**Results:**

After a median follow-up of 14.24 years (95% CI [14.21–14.25]), 93 colorectal cancer cases were observed. This is equivalent to a truncated age-standardized rate of 69.0 (95% CI [43.3–94.7]) for male and 43.4 (95% CI [29.4–57.5]) for female participants (≥ 50 years at colonoscopy). The ratio of this observed to the expected rate from cancer registry data showed a 67% decrease in colorectal cancer incidence in the male and 65% in the female participants (*p* < 0.0001). In multivariate analysis of screening patients, age at screening (*p* < 0.0001) was the main predictive factor for colorectal cancer. In the subgroup with positive polyp findings, age (*p* < 0.0001) and the polyp size (*p* = 0.0002) were associated with colorectal cancer.

**Conclusion:**

These results underline the significance of a full colonoscopy screening combined with polypectomy in reducing the total disease burden of colorectal cancer.

**Supplementary Information:**

The online version contains supplementary material available at 10.1007/s00432-021-03532-7.

## Introduction

Although colorectal cancer (CRC) rates have declined in the last decade, it remains among the most frequently diagnosed cancers. Therefore, screening programs with high patient participation rates are essential in decreasing the overall disease burden and mortality from CRC through early detection of neoplasms or removal of precursor lesions. A large number of countries have recently adopted colonoscopy for the general population as the primary screening instrument for CRC. This screening procedure has consistently shown a high sensitivity and also provides a simultaneous curative polyp removal (Lieberman 2009; Issa and Noureddine 2017). In Germany, screening colonoscopy within a quality assurance program has been offered since 2002. However, overall participation and adherence remains relatively low at 58.5% (Starker et al. 2018). An organized screening began in 2019 with invitations for subjects aged ≥ 55 years (no upper age limit) and with the option of a second screening colonoscopy ≥ 10 years later if no polyps are found. Persons with increased risk factors such as a familial history of CRC or previous history of inflammatory bowel disease may be screened earlier (Niedermaier et al. 2018; AWMF 2019).

Studies detailing the impact of colonoscopy on cancer incidence or mortality have mainly been designed as observational cohort or case–control studies because of the already proven efficacy of sigmoidoscopy in reducing cancer incidence. Most published studies have reported a drop in both incidence and mortality mainly attributed to polypectomy, most notably the National Polyp Study which reported a 76% decrease in CRC incidence (Winawer et al. 1993; Thiis-Evensen et al. 1999; Kaminski et al. 2010; Zauber et al. 2012). Two large RCTs by Atkin et al. and the PLCO by Schoen et al. using sigmoidoscopy, limited to an examination of the distal colon, reported a CRC incidence reduction of 21% and 23% compared to a control group not screened (Atkin et al. 2010; Schoen et al. 2012). Comparisons to non-invasive screening tests such as the fecal immunochemical test showed an increase in patient participation, while the detection rate of precursor lesions was lower (Quintero et al. 2012).

The objective of the present study was therefore to determine the impact of a colonoscopy screening examination on the colorectal cancer incidence of a regional cohort of participants within the scope of the Munich Cancer Registry (MCR) in Bavaria, Germany.

## Methods

### Study participants

A closed cohort of participants was included in a prospective observational study following a singular colonoscopy exam with or without polypectomy. The study design was observational and did not include any procedures for blinding or randomization. Only a single colonoscopy examination was documented in the study, and information on additional examinations, as recommended in current guidelines, was not available. In addition, information on the indication of the colonoscopy was not documented and may have been both for purely screening purposes and due to apparent symptoms. Participants were eligible for the study if they underwent a colonoscopy examination at one of the 48 participating gastroenterology practices within the scope of the MCR (Supplemtary Table 2). The catchment area of the MCR currently encompasses a population of approximately 4.9 million inhabitants Munich Cancer Registry (MCR) (2019). An informed consent was obtained from all study participants prior to the examination. The study conduct followed the tenets of the Declaration of Helsinki. For each participant, baseline demographic and colonoscopy data were collected, namely all excised polyps were morphologically categorized as adenomatous, non-adenomatous, or mixed type. Maximum size of the polyp diameter (measured using a ruler by the examining pathologists) and quantity were also recorded.

**Fig. 1:**
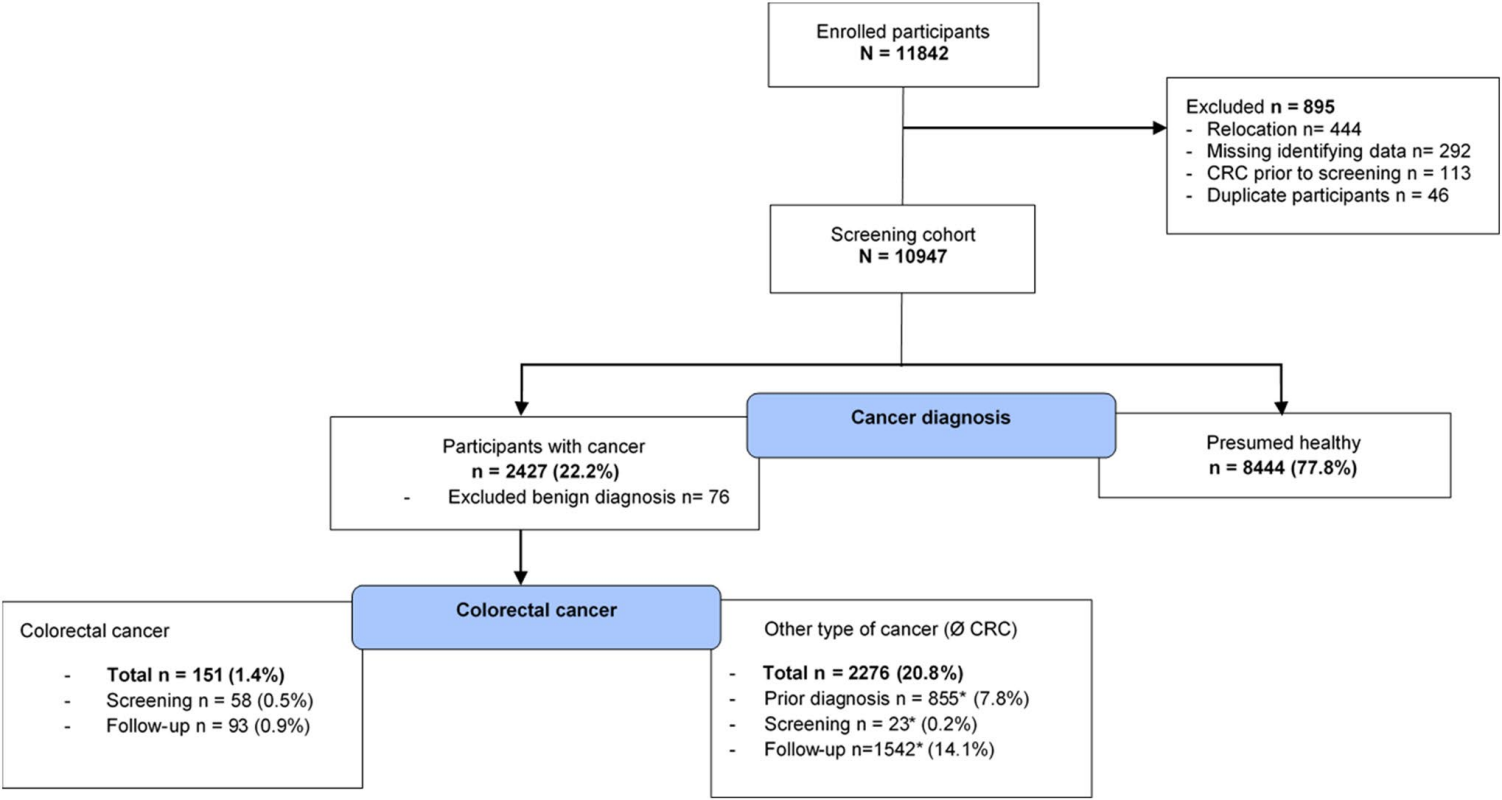
Study participant flowchart: CRC, colorectal cancer. Percentages are derived from the total screening cohort. *Indicates multiple cancer diagnoses counted separately for each time point. Ø, without

By follow-up data linkage with MCR data, subsequent or prior cancer diagnoses were merged to the dataset of participants, with a final data retrieval conducted in July 2020 and final life status follow-up in Mai 2019. A total of 11,842 participants were recruited for the study between January 2005 and September 2009 after a quality assurance program was established. Excluding participants with missing identifying data (*n* = 292), duplicate screening data (*n* = 46), prior colorectal tumor diagnoses (*n* = 113), or moving out of the MCR catchment area (*n* = 444) resulted in a final study cohort of 10,947 participants. Benign (*n* = 76) diagnoses were excluded and 58 colorectal cancer diagnoses during the procedure or within the first 30 days following the screening date were considered separately. A total of 93 CRC cases were observed after a median follow-up of 14.24 years. Figure 1 shows the participant flowchart.

### Statistical analysis

Assuming a power of 80% and a type I error of 5%, the sample size calculation was based on the estimation that 0.16% of the population at 60 years old would develop CRC. Considering a 5% loss to follow-up quota and 75% negative colonoscopy findings, a required sample size of 8000 participants was determined.

The primary outcome of this study was to determine the impact of the colonoscopy examination on colorectal cancer incidence during the follow-up observation period. Cancer incidence in the study cohort was determined using the person-years at risk per sex and 5-year age groups, adjusted to BRD standard to obtain the ASR (age-standardized rate), as well as the truncated age-standardized rate in the designated screening population (TASR, age > 50 years at screening). Although the recommended screening age is currently set at 55 in women the cut-off age 50 years was used for both sexes due to the ongoing discussion on a general lower age limit for the initial colonoscopy screening (Vuik et al. 2019). This was performed for the screening study cohort and for a population-based cohort from the MCR during the same time period. The standardized incidence ratios for incidence were used to compare rates. Poisson distribution was assumed for the number of CRC cases and the 95% confidence interval was calculated accordingly. The cumulative risk was calculated according to Bray et al. (2017).

Time to CRC data for the primary outcome was calculated from the date of the screening procedure (*n* = 10,947) until the first colorectal cancer diagnosis. To account for the competing risk of death by any cause, a cumulative incidence analysis was used to calculate the time to CRC. Differences among subgroups were assessed using Fine’s Gray Test for Equality of Cumulative Incidence Functions (Kalbfleisch and Prentice 2011). The subgroup of participants considered presumably healthy without any recorded tumor diagnosis (CRC or other cancer type) were not consistently monitored and no follow-up data were available. Therefore, the expected survival time was estimated using national life tables.

Overall survival was determined for participants with prevalent and incidental CRC. Overall survival (OS) from date of CRC until date of death was estimated using the Kaplan–Meier method and tested using the log-rank test.

For a graphical comparison of OS, five age- and sex-matched random samples were drawn from the MCR population during the same time period: four samples corresponding to the sample size of incidental CRC cases (samples 1–3), as well as one larger sample (Max cohort MCR—follow-up). One sample corresponds to the prevalent cases (Max cohort MCR—screening).

A multivariate analysis of independent predictive factors were assessed using the Fine‐Gray sub-distribution hazard model (Fine and Gray 1999). The hazard ratio and 95% CIs are given in the results. Baseline demographics and colonoscopy results were entered simultaneously as independent predictive variables for a multivariate analyses of CRC risk. Similar to the calculation of CRC incidence, only patients > 50 years at screening were included.

Pearson’s chi-square was used to compare categorical, and Student’s *t* test for numerical variables between individual subgroups. The Pearson’s correlation coefficient was used to determine bivariate correlation of continuous variables. Percentages for individual subgroups consider available data only, and missing values are given in relation to the underlying cohort or subgroup for the respective category.

For all analyses, a two-sided *p* value of 0.05 or less was considered statistically significant. IBM SPSS Statistics version 25 and Statistical Analysis Software version 9.4 (SAS Institute, Cary, NC) were used for data analysis.

## Results

### Screening demographics

Baseline demographics of the screening cohort are shown in Table 1. The study participants had a median age of 62 years at screening (range 15–97) and therefore within the recommended age range for colonoscopy screening.

**Table 1:** Baseline cohort demographics and screening characteristics, overall, and for male and female subgroups individually

	*N* (%)	Person-years	CRC cases (% of screening cohort)	Cases per 100,000 prs.years	Male *n* (%)	Female *n* (%)	*p* value
Total	10 947	150,714.1	93 (0.9)	61.7			
Sex							
Male	4851 (43.3)	65,572.8	48 (1.0)	73.2			
Female	6096 (55.7)	85,141.3	45 (0.7)	52.9			
Age groups [years at screening]							
< 50	1240 (11.3)	17,457.8	1 (0.1)	5.7	538 (11.1)	702 (11.5)	< 0.0001
50–59	2964 (27.1)	42,163.5	13 (0.4)	30.8	1176 (24.2)	1788 (29.3)	
60–69	4924 (45.0)	69,752.0	42 (0.8)	60.2	2260 (46.6)	2664 (43.7)	
70–79	1623 (14.8)	19,952.2	35 (2.2)	175.4	785 (16.2)	838 (13.8)	
≥ 80	196 (1.8)	1388.6	2 (1.0)	144.0	92 (1.9)	104 (1.7)	
Colonoscopy result							
No finding	6820 (62.3)	94,554.6	46 (0.6)	48.7	2267 (55.0)	4153 (68.1)	< 0.0001
Polyps found	4127 (37.7)	56,159.5	47 (1.1)	83.7	2184 (45.0)	1943 (31.9)	
Polyp histology							
Adenomatous	2536 (61.4)	34,352.8	30 (1.2)	87.3	1386 (63.5)	1150 (59.2)	< 0.0001
Non-adenomatous	1173 (28.4)	16,193.6	9 (0.8)	55.6	555 (25.4)	618 (31.8)	
Mixed adenomatous/non-adenomatous	418 (10.1)	5613.2	8 (1.9)	142.5	243 (11.1)	175 (9.0)	
Number of polyps [*n*]							
Adenomatous							
1–2	1950 (78.8)	26,625.7	17 (0.9)	63.8	1023 (75.7)	927 (82.6)	< 0.0001
3–4	407 (16.4)	5420.1	13 (3.2)	239.8	246 (18.2)	161 (14.3)	
≥ 5	118 (4.8)	1557.8	0	–	83 (6.1)	35 (3.1)	
Missing	61 (2.4)	–			34	27	
Non-adenomatous							
1–2	1007 (87.7)	13,940.5	8 (0.8)	57.4	466 (86.5)	541 (88.8)	0.457
3–4	96 (8.4)	1326.0	0	–	49 (9.1)	47 (7.72)	
≥ 5	45 (3.9)	610.4	0	–	24 (4.5)	21 (3.45)	
Missing	25 (2.1)	–			16	9	
Mixed adenomatous/non-adenomatous^a^							
1–2	298 (71.3)	3957.6	8 (2.7)	202.1	173 (71.2)	125 (71.4)	0.564
3–4	90 (21.5)	1249.6	0	–	50 (20.6)	40 (22.9)	
≥ 5	30 (7.2)	406.0	0	–	20 (8.2)	10 (5.7)	
Largest polyp diameter [mm]							
Adenomatous							
< 5	835 (34.2)	11,438.7	5 (0.6)	43.7	447 (33.5)	388 (35.1)	0.129
5–10	1202 (49.2)	16,426.8	9 (0.7)	54.8	649 (48.6)	553 (50.0)	
> 10	405 (16.6)	5203.4	15 (3.7)	288.3	240 (18.0)	165 (14.9)	
Missing	94 (3.7)	–	–	–	50	44	
Non-adenomatous							
< 5	659 (58.4)	9111.2	8 (1.2)	87.8	317 (60.3)	342 (56.8)	0.365
5–10	430 (38.1)	5945.8	1 (0.2)	16.8	194 (36.9)	236 (39.2)	
> 10	39 (3.5)	545.0	0	–	15 (2.8)	24 (4.0)	
Missing	45 (3.8)	–	–	–	29	16	
Mixed adenomatous/non-adenomatous^a^							
< 5	145 (35.6)	1971.7	3 (2.1)	151.2	81 (34.2)	64 (37.4)	0.756
5–10	209 (51.4)	2758.4	3 (1.4)	108.8	125 (52.7)	84 (49.1)	
> 10	54 (13.0)	754.0	1 (1.8)	132.6	31 (13.1)	23 (13.5)	
Missing	10 (6.0)	–	–	–	6	4	

Percentages for individual subgroups consider available data only, and missing values are given in relation to the underlying cohort or subgroup for the respective category

^a^The maximum value from either adenomatous or non-adenomatous was selected. *p* values calculated with *χ*^2^ test to compare male and female subgroups

### Colonoscopy

In the majority of participants, no polyps were found (62.3%, Table 1). In the subgroup with manifest polyps, the histology was mainly adenomatous (61.4%), 28.4% were classified as non-adenomatous, and the remaining polyps (10.1%) were categorized as having mixed adenomatous/non-adenomatous histology. The median number of polyps varied between one in the adenomatous (range 1–101) and non-adenomatous (range 1–100) groups, and two in the mixed group (range 1–20). The adenomatous and mixed polyps both had a median maximum diameter of 5 mm (adenomatous, range 1–200 mm; mixed, range 1–80 mm), followed the non-adenomatous subgroup (Median = 4 mm, range 1–40 mm). Age at screening was correlated to size (*p* = 0.0175) but not to the number of polyps found (*p* = 0.4092). The number of polyps and maximum size were also correlated (*p* < 0.0001).

### Sex-based differences

Slightly more female (55.7%) than male participants took part in the study (*p* < 0.0001). This sex-based discrepancy was especially evident for participants between 50 and 59 years (female 60.3% vs. 39.7%), but remained consistent over all other age groups, as well. Female participants were also on average 1 year younger (median 62.0 vs. 63.0). Sex-based differences were also observed in the colonoscopy findings with fewer polyps found in female participants compared to male participants (*p* < 0.0001). Female participants also presented more frequently with non-adenomatous (31.8% vs. 25.4%) compared to their male counterparts, while overall size and number was similar. Female participants with adenomatous polyp histology had fewer polyps in total (*p* < 0.0001).

### CRC prevalence

In total, 151 participants were diagnosed with colorectal cancer, 58 during the screening colonoscopy. These 58 cases are equivalent to three-times the expected incidence in this cohort. These participants were a median 68 years (range 43.0–89.0) at diagnosis and predominately male (65.5%) (Table 2). Tumors were mainly found in the left colon (79.3%), with an intermediate grade (77.6%) and adenocarcinoma histology (93.1%) characterizing the tumor biology. A total of 52.7% of cases presented in UICC stages I or II.

**Table 2:** Colorectal cancer during screening and follow-up period

	Prevalent CRC			Incidental CRC		
	*n* (%)	Male *n* (%)	Female *n* (%)	*n* (%)	Male *n* (%)	Female *n* (%)
Participants with CRC	58	38	20	93	48	45
Age [years at diagnosis]						
40–44	4 (6.9)	2 (5.3)	2 (10.0)	0	0	0
45–49	1 (1.7)	1 (2.6)	0	0	0	0
50–54	2 (3.4)	1 (2.6)	1 (5.0)	1 (1.1)	1 (2.1)	0
55–59	3 (5.2)	2 (5.3)	1 (5.0)	1 (1.1)	0	1 (2.2)
60–64	9 (15.5)	5 (13.2)	4 (20.0)	10 (10.8)	6 (12.5)	4 (8.9)
65–69	13 (22.4)	9 (23.7)	4 (20.0)	12 (12.9)	5 (10.4)	7 (15.6)
70–74	11 (19.0)	8 (21.0)	3 (15.0)	20 (21.5)	11 (22.9)	9 (20.0)
75–79	9 (15.5)	5 (13.2)	4 (20.0)	26 (28.0)	11 (22.9)	15 (33.3)
80–84	4 (6.9)	3 (7.9)	1 (5.0)	14 (15.1)	9 (18.8)	5 (11.1)
≥ 85	2 (3.4)	2 (5.3)	0	9 (9.7)	5 (10.4)	4 (8.9)
Side						
Left	46 (79.3)	32 (84.2)	14 (70.0)	41 (44.1)	24 (50.0)	17 (37.8)
Right	12 (20.7)	6 (15.8)	6 (30.0)	52 (55.9)	24 (50.0)	28 (62.2)
Localization						
Colon	30 (51.7)	17 (44.7)	13 (65.0)	67 (72.0)	37 (77.1)	30 (66.7)
Rectosigmoid junction	10 (17.2)	7 (18.4)	3 (15.0)	5 (5.4)	3 (6.2)	2 (4.4)
Rectum	18 (31.0)	14 (36.8)	4 (20.0)	21 (22.6)	8 (16.7)	13 (28.9)
UICC stadium^a^						
0	0	0	0	2 (2.2)	2 (4.8)	0
I	21 (36.2)	10 (26.3)	11 (55.0)	24 (25.8)	13 (31.0)	11 (27.5)
II	8 (13.8)	6 (15.8)	2 (10.0)	29 (31.2)	14 (33.3)	15 (37.5)
III	19 (32.7)	15 (39.5)	4 (20.0)	18 (19.4)	8 (19.0)	10 (25.0)
IV	7 (12.1)	6 (15.8)	1 (5.0)	9 (9.7)	5 (11.9)	4 (10.0)
Missing	3 (5.2)	1 (2.6)	2 (10.0)	11 (11.8)	6	5
Grading						
G1	1 (1.7)	1 (2.6)	0	8 (8.6)	4 (10.3)	4 (9.5)
G2	45 (77.6)	31 (81.6)	14 (70.0)	56 (60.2)	30 (76.9)	26 (61.9)
G3	9 (15.5)	6 (15.8)	3 (15.0)	17 (18.3)	5 (12.8)	12 (28.6)
Missing	3 (5.2)	0	3 (15.0)	12 (12.9)	9	3
Histology						
Adenocarcinoma	54 (93.1)	36 (94.7)	18 (90.0)	64 (68.8)	32 (76.2)	32 (76.2)
Mucinous carcinoma	2 (3.4)	2 (5.3)	0	7 (7.5)	4 (9.5)	3 (7.1)
Carcinoma in situ	2 (3.4)	0	2 (10.0)	6 (6.5)	4 (9.5)	2 (4.8)
Carcinoid tumor	0	0	0	4 (4.3)	2 (4.8)	2 (4.8)
Signet ring cell carcinoma	0	0	0	2 (2.2)	0	2 (4.8)
GIST	0	0	0	1 (1.1)	0	1 (2.4)
Missing	0	0	0	9 (9.7)	6	3
CRC mortality^b^						
Alive	32 (55.2)	15 (39.5)	17 (85.0)	65 (69.9)	33 (68.8)	32 (71.1)
Deceased	26 (44.8)	23 (60.5)	3 (15.0)	28 (30.1)	15 (31.3)	13 (28.9)

Percentages for individual subgroups consider available data only, and missing values are given in relation to the underlying cohort or subgroup for the respective category

^a^UICC stadium according to TNM classification version 8

^b^Last date of life status follow-up Mai, 2019

### CRC incidence

A total of 93 CRCs occurred in the follow-up observation period (Table 2). Cases were observed in men (51.6%) and in older participants irrespective of sex (overall median age at diagnosis = 75, range [52–92]). The majority of tumors were located on the right side of the colon (55.9%) and presented in the more favorable UICC stages I or II (57.0%). The tumor histopathology was primarily intermediate grade adenocarcinoma. The CRC diagnosis represented the first tumor diagnosis for most participants (77.4%), 19.4% and 3.2% of participants had one or two previous cancer diagnoses not located in the colon. The events observed in the study screening participants resulted in an ASR of 24.1 (95%CI [15.3–33.0]) for male and 14.8 (95%CI [10.1–19.6]) for female participants (Supplementary Table 1). The expected values were derived from the equivalent MCR data and yielded an ASR of 73.4 (95%CI [72.2–74.6]) for the male and 45.2 (95%CI [44.3–46.0]) for the female population. The standardized incidence ratio between both (SIR) was 0.33 for men (95%CI [0.27–0.40]), and 0.33 for women (95%CI [0.28–0.39]; *p* < 0.0001).

Limiting the comparison to the population of interest in a truncated analysis (screening age > 50 years) resulted in a truncated age-standardized rate (TASR) of 69.0 (95%CI [43.3–94.7]) in male and 43.4 (95%CI [29.4–57.5]) in female participants. The respective TASR in the MCR population was 208.4 (95%CI [204.9–211.8]) in the male and 124.1 (95%CI [121.8–126.5]) in the female population. A comparison of both TASR rates showed a ratio of 0.33 (95%CI [0.27–0.40]) and 0.35 (95%CI [0.29–0.42]), respectively, for men and women (*p *< 0.0001, Supplementary Table 1).

This number of recorded events was equivalent to a cumulative incidence of 0.3 (95%CI [0.2–0.5]) for men and 0.2 (95%CI [0.1–0.4]) for women after 5 years of follow-up, which is equivalent to, respectively, 32% of the total observed CRC cases. The overall cumulative risk for CRC came to 4.6% for men and 2.3% for women in the study cohort compared to a cumulative risk of 10.2% and 6.8% in the comparison populations.

### CRC incidence by sex and polyps

**Fig. 2:**
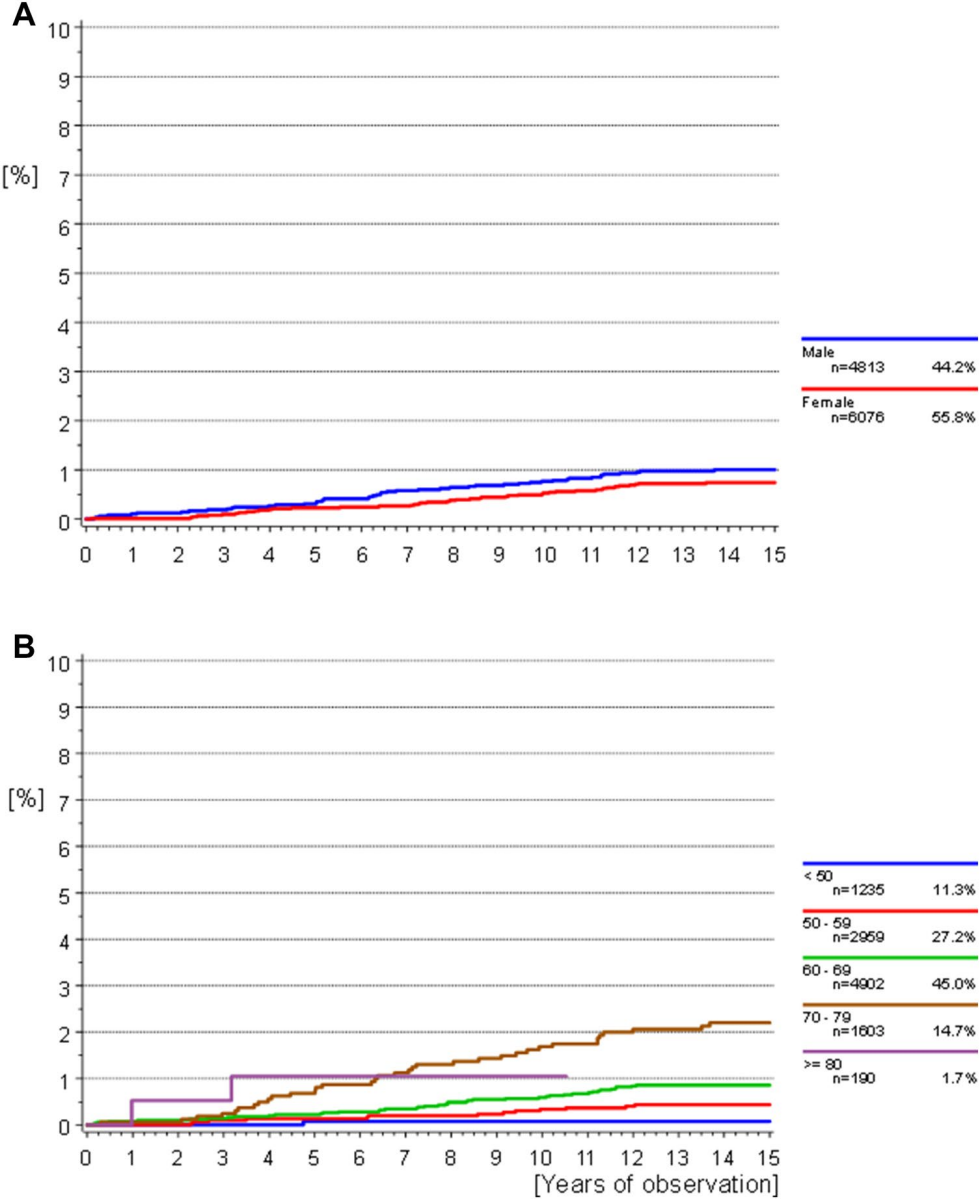
**a**, **b** Cumulative incidence of CRC: **a** starting from initial screening date according to sex. Competing risk death without CRC considered. Gray’s test *p* = 0.1473. **b** According to age group at screening. Competing risk of death without CRC considered. Gray’s test *p* < 0.0001

**Fig. 3:**
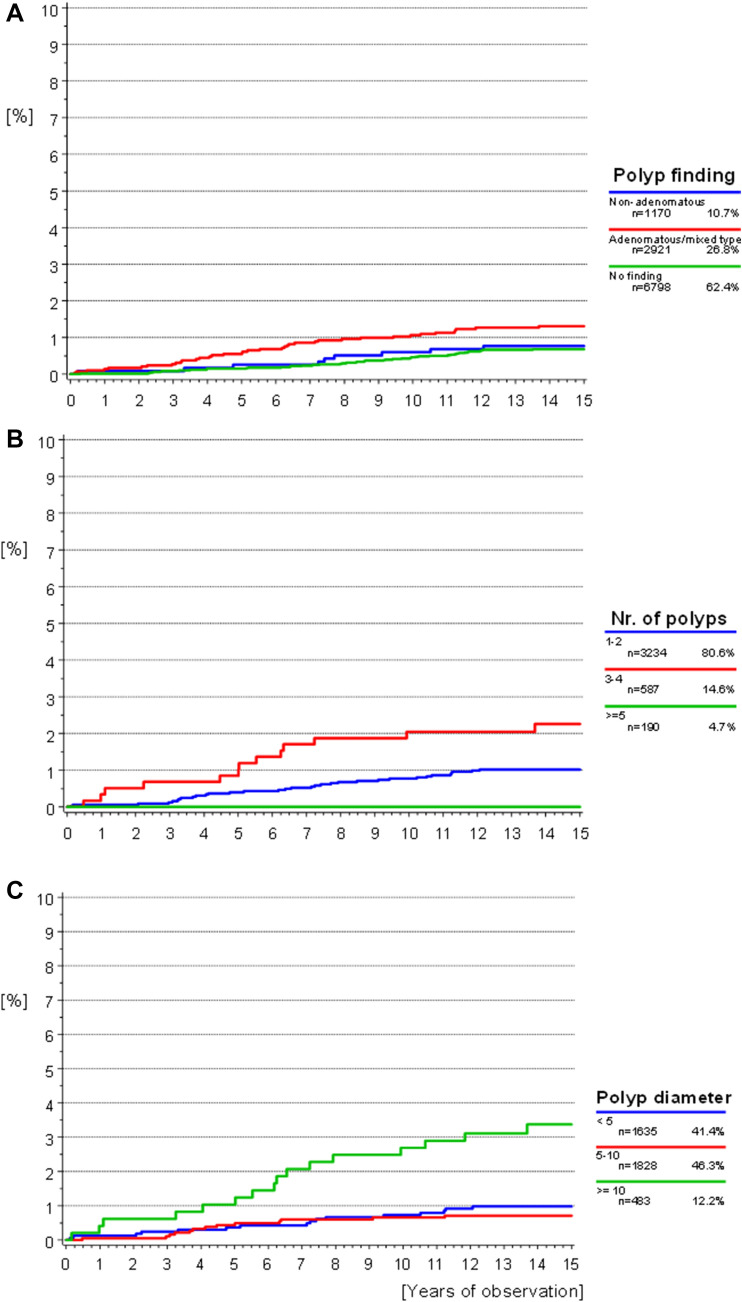
**a**–**c** Cumulative incidence of CRC: **a** according to screening findings. Polyps with adenomatous components combined into one group versus non-adenomatous polyps or inconspicuous findings. Gray’s test *p* = 0.0084. **b** According to polyp quantity. Gray’s test *p* = 0.0137. **c** According to the maximum polyp diameter. Gray’s test *p* < 0.0001

The median follow-up time was 14.24 years (95%CI [14.21–14.25] years). Median time from screening to diagnosis was 6.4 years in men (range 0.2–13.7 years) and 7.9 years in women (range 0.1–13.5 years, Fig. 2a). Out of all cases, a CRC diagnosis was made within the first 6 months after the study colonoscopy in five men and one woman. Figure 2a illustrates the cumulative CRC incidence over the entire observation period stratified by sex. Although there were slightly more events observed for male participants, this difference was not significant (*p* = 0.1473). The cumulative incidence according to age did show a significant difference; here, older participants (> 70 years at screening) showed the highest incidence of CRC (*p* < 0.0001). Polypectomy results (*p* = 0.0084), as well as the number (*p* = 0.0137) and maximum diameter of removed polyps (*p* < 0.0001) likewise influenced the cumulative CRC incidence. A risk for CRC remained even in patients without any detected polyps. Polyps with an adenomatous component, numbering 3–4 polyps, and a maximum diameter of ≥ 10 mm were most highly associated with a subsequent CRC diagnosis (Fig. 3a–c).

### Predictive factors and prognosis

In a multivariate fine-gray model of the factors sex (*p* = 0.4521), polyp finding (*p* = 0.0988), only age at diagnosis remained as a significant independent predictor for CRC (*p* < 0.0001, Table 3). Focusing on the participants with polyps in a model with sex, age, and polyp characteristics summarized in Table 3, again age was an independent predictor of CRC (*p* < 0.0001), as well as the maximum polyp diameter (*p* = 0.0002). Polyp histology (*p* = 0.5200), sex (*p* = 0.6701), and the number of polyps (*p* = 0.1030) were not significant in the model.

**Table 3:** Multivariate fine-gray model of predictive factors for diagnosis of colorectal cancer during follow-up

Variable	Groups	Hazard ratio	95% CI	*p* value
Model_Study_ [*n* = 9654]				
Age at screening				< 0.0001
	50–59	Ref.	–	–
	60–69	1.862	1.004–3.454	0.0484
	70–79	4.688	2.506–8.770	< 0.0001
	≥ 80	2.349	0.530–10.404	0.2607
Sex				0.4521
	Female	Ref.	–	–
	Male	1.171	0.776–1.776	0.4521
Polyp finding				0.0988
	No finding	Ref.	–	–
	Adenomatous/mixed	1.564	1.017–2.403	0.0415
	Non-adenomatous	0.947	0.447–2.007	0.8878
Model_Polyp_ [*n* = 3467]				
Age at screening				< 0.0001
	50–59	Ref.	–	
	60–69	2.796	0.821–9.521	0.1000
	70–79	9.997	3.013–33.169	0.0002
	≥ 80	5.364	0.556–51.766	0.1471
Sex				0.6701
	Female	Ref.		–
	Male	1.141	0.621–2.098	
Polyp histology				0.5200
	Non-adenomatous	Ref.		
	Adenomatous	1.127	0.514–2.471	0.7660
	Mixed	1.772	0.599–5.247	0.3016
Polyp N^a^				0.1030
	1–2	Ref.		–
	3–4	1.717	0.896–3.289	0.1030
Polyp mm				0.0002
	< 5	Ref.		
	5–10	0.737	0.351–1.550	0.4215
	≥ 10	3.110	1.538–6.291	0.0016

The model_Study_ was constructed using all screening study participants older than 50 years at screening without a diagnosis of CRC during screening. The model_Polyp_ furthermore only considered participants over 50 years at screening with polyp findings

^a^No event in the subgroup of participants with a maximum of  ≥ 5 polyps

**Fig. 4:**
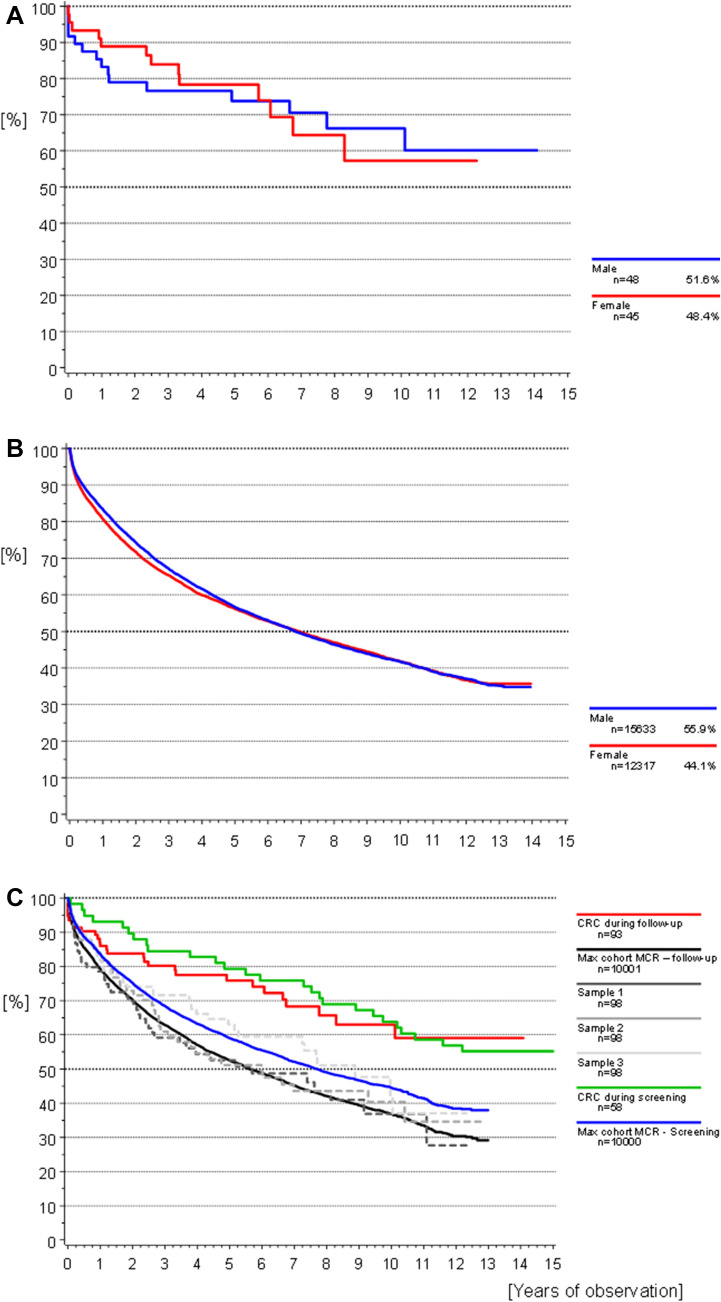
**a**–**c** Overall survival: **a** participants with CRC during follow-up stratified by sex. Log-rank univariate *p* = 0.9821. **b** MCR population with CRC during the same time period as the study stratified by sex. **c** Participants with CRC in comparison to five randomly drawn age- and sex-matched samples from the MCR population data

At the last date of follow-up 69.9% of participants with CRC diagnosis were still alive. Comparison of overall survival after CRC according to sex was not significant, most likely due to the small sample size and large data variance (Fig. 4a *p* = 0.9821). In Fig. 4b, the overall survival of the MCR population from the same time period is shown. Figure 4c compares the OS of the study cohort (incidental and prevalent cases) to that of five representative random samples from the MCR population data. The overall 3-year survival of the incidental CRC cases was 80.2% and the 5-year survival rate was 75.9%, compared to 66.3/55.8% in the MCR population. Median survival time was 7.68 years (95% CI [6.25–10.67]), 6.76 (95%CI [4.96–9.20]) in men and 5.90 years in women, respectively (95%CI [3.71–7.92]; Fig. 2a).

## Discussion

The study detailed above aimed to examine the association between colonoscopy screening and subsequent CRC incidence and prognosis in a cohort of participants from Bavaria, Germany. Although a broad range of age groups were included in the study data, the final analysis focused on participants 50 years and older. This population is most at risk for CRC and therefore benefits the most from taking part in a screening colonoscopy program. A comparison of the CRC incidence to a matched population from the same region showed a significant drop in incidence among the screening target participants of 67% and 65% in men and women, respectively. The main predictive factor for CRC in univariate and multivariate analyses was age at screening, and for participants with polypectomy, a maximum diameter of over 10 mm was also indicative for CRC. These findings are concurrent to and supported by findings from a large systematic review by Brenner et al. published 2014 (Brenner et al. 2014), as well as other more recent studies which reported a similar drop in CRC incidence (Pan et al. 2016; Ren et al. 2016; Click et al. 2018; Helsingen et al. 2019; Lee et al. 2020). The finding that polyp size is predictive for future CRC diagnosis further underscores the significance of this factor, especially considering its previously reported association to a less favorable histology and long-term risk of CRC (Pickhardt et al. 2018). The 10 mm cut-off represents an especially critical clinical factor, since a small percentage of these polyps contain cancerous cells (Lieberman 2009; Parsa et al. 2019). While a large study by Lee et al. or by He et al., as well as older studies found a similar association, the current study replicates these findings in a more recent, prospective cohort of participants not limited to sigmoidoscopy (Atkin et al. 1992; He et al. 2020; Lee et al. 2020). Prognosis after CRC was also improved in a comparison to matched data samples from the MCR. Although survival in the prevalent CRC subgroup was similar to that of the cases during the follow-up period, this may largely be attributed to a younger age at diagnosis in the prevalent subgroup. An interesting finding was also the shift from the mainly left-sided CRC in the prevalent group to the slightly more frequent right-sided cases observed during follow-up. Similar results were found in previous studies (Brenner et al. 2010). Reasons for this phenomenon may be that polyps on the right side tend to be harder to detect and removed during colonoscopy due to their smaller size and more flattened appearance (Gupta et al. 2012). Other aspects such as bowel preparation may also play a role.

Looking at screening participation more women than men took part in the procedure. Women also had fewer positive findings, and polyps were mainly associated with a better prognosis compared to men. This sex-based difference in health-seeking behavior and colonoscopy findings are similar to results found in other studies with a similar objective (Gimeno Garcia 2012; Navarro et al. 2017). However, when taking age into consideration, no effect of sex remained.

The quality of the colonoscopy procedure is also frequently discussed in playing an important role in screening efficacy. Fast proliferating de-novo tumors or interval cancers may be missed during a colonoscopy, the rate has been reported to lie between 2 and 6% (Samadder et al. 2014). In addition, an incomplete resection may also lead to a subsequent CRC; here, the reported rate from the CARE study was determined to lie at approximately 10% (Pohl et al. 2013). The interval cases in this study cannot be identified and are therefore not classified as such; their impact can therefore not be determined. However, these are found in all screening-associated studies and irrespective of methodologies and compared to sporadic cases of CRC prognosis was nearly equivalent (Erichsen et al. 2013). According to a study by Ertem et al. in 2018, physicians performing colonoscopies will only fail to detect 2–4 interval CRCs during a 35-year practice (Ertem et al. 2018). This indicates that these missed tumors only have a minor impact on the overall number of cases.

Limiting the validity of these observed effects are the observational nature of this study and its limited strength in assessing causal association. Considering lead-time bias, the results above may falsely represent a better prognosis compared to the general population who may or may not have undergone the same examination. Therefore, any improvement in survival may be due to an earlier detection and generalizations should be made with caution (Facciorusso et al. 2016). Also, an overdiagnosis bias may have selectively detected small and slower growing polyps rather than fast growing ones. However, which polyp will differentiate into an invasive carcinoma cannot be predicted and any observed effect can therefore not be fully attributed to the screening examination itself.

Aside from general limitations common to all screening studies, a main limitation in the study data is the lack of information on the reason for partaking in the examination. It may be assumed that not all participants can be considered asymptomatic screening patients, but were instead referred to the exam by their primary care physician based on reported symptoms. Therefore, the outcome of the analyses may more likely reflect the effect of the polypectomy procedure studied for example by the National Polyp Study of 1993 by Winwar et al. (1993), which may be more suitable for direct comparison. Here, the authors also found a large reduction in CRC incidence following polypectomy during one or more colonoscopies over the study period. In addition, the interval for excluded cases of CRC after colonoscopy should have encompassed a longer time span to definitely rule out any missed carcinomas. Although only six cases occurred between 30 days and 6 months after the colonoscopy, it is unclear whether they developed de-novo or not.

Regardless of the evaluated methodology, colonoscopy examinations find, and a polypectomy removes precursor lesions, and both have a proven positive effect on CRC risk. Especially, adenomatous polyps are at an increased risk for developing into adenocarcinomas, slightly over half of polyps found during a colonoscopy were of this type (Jass et al. 2006). The reported percentages were similar to those found in the study data, and in univariate analysis significantly associated with subsequent CRC diagnosis. Comparison of adenomatous and mixed adenomatous/non-adenomatous polyps showed that an adenomatous component caused the polyp to carry a similar risk for CRC as those solely characterized as adenomatous. Non-adenomatous polyps on the other hand are found less frequently and carry almost no malignancy risk (Harken and Moore 2018). In the study data, the participants with this type of polyp showed a risk for CRC comparable to those without any findings (Fig. 3a). However, polyp histology was not significant in multivariate analysis, indicating that age-related changes to the colon microenvironment or microbiome may play a more influential role on carcinogenesis. Interestingly, the number of polyps was also only significant in univariate analysis; however, the subgroup with the highest number of polyps only represented a small number of participants and no cases of CRC. Potentially, a longer follow-up would increase the number of events in this category and a significant association would be found as expected. This factor is considered a main quality indicator for the colonoscopy procedure and was associated with subsequent CRC in other studies (Lieberman et al. 2000; Amano et al. 2018; Lee et al. 2018). If no polyps were found, a risk for CRC remained. In these participants without any polyp findings, the reason for CRC development could be an underlying inflammatory disease, fast growing interval cancers, or the quality of the colonoscopy procedure. These findings are similar to the published data by He et al. where advanced polyps also represented a higher risk in regard to CRC development, participants without polyps also showed a lowered but inherent CRC risk (He et al. 2020). Regardless of polyp findings, participants with a successful colonoscopy procedure will likely seek follow-up screening and thus lower their risk for CRC in the long run.

No additional histopathological data on the removed polyps were collected to further refine patient risk stratification. Potentially, more detailed pathological classification of polyps could further refine patient subgroups and determine who would profit from more frequent surveillance exams.

In conclusion, colonoscopy with polypectomy is an effective screening tool in reducing the overall number of CRC in the general population. The incidence of CRC is expected to increase accordingly and with it the associated disease burden. A high uptake of preventive colonoscopy screening would therefore decrease the overall number of expected cases, especially those induced through the adenoma–carcinoma sequence. Fewer cases would mean alleviating some of the rising costs estimated to overwhelm the health care system in the next 10 years (Keum and Giovannucci 2019). A risk-based and adjusted screening interval for patients with larger polyp findings could be easily implemented and prove highly beneficial. The results of the study indicate that, in particular, younger participants may benefit especially, considering that larger polyps could be removed early in their growth and thereby lower the risk for CRC in the following years. Therefore, increasing participation and adherence to screening programs should be a priority considering the expected increase in cases due to an increasingly aging population (Keum and Giovannucci 2019).

## Supplementary Information

Below is the link to the electronic supplementary material.

Supplementary file1 (PDF 102 KB)

## Data Availability

Study data can be made available for scientific purposes upon request and only after approval through study lead.

## Code availability

Analysis code can also be made available upon request.
