# Supplementary material for: Colonoscopy and polypectomy: beside age, size of polyps main factor for long-term risk of colorectal cancer in a screening population
Source: J Cancer Res Clin Oncol. 2021 Feb 4;147(9):2645–58. doi: 10.1007/s00432-021-03532-7 (PMC8310861; doi:10.1007/s00432-021-03532-7)
Supplement: Supplementary file 1 — Supplementary file1 (PDF 102 KB) [file 432_2021_3532_MOESM1_ESM.pdf]

Title: Colonoscopy and polypectomy: Beside age, size of polyps main factor for long-term risk of colorectal cancer in a screening population

Journal: Journal of Cancer Research and Clinical Oncology

Authors:

Halfter Kathrin, Dr.

Munich Cancer Registry (MCR), Institute for Medical Information Processing, Biometry and Epidemiology, Ludwig-Maximilians-University (LMU), Marchionini Str. 17, 81377 Munich Germany

Bauerfeind Lea

Munich Cancer Registry (MCR), Institute for Medical Information Processing, Biometry and Epidemiology, Ludwig-Maximilians-University (LMU), Marchionini Str. 17, 81377 Munich Germany

Schlesinger-Raab Anne, Dr.

Munich Cancer Registry (MCR), Institute for Medical Information Processing, Biometry and Epidemiology, Ludwig-Maximilians-University (LMU), Marchionini Str. 17, 81377 Munich Germany

Schmidt Michael, Staatsexamen

Munich Cancer Registry (MCR), Institute for Medical Information Processing, Biometry and Epidemiology, Ludwig-Maximilians-University (LMU), Marchionini Str. 17, 81377 Munich Germany

Schubert-Fritschle Gabriele, Dr.

Munich Cancer Registry (MCR), Institute for Medical Information Processing, Biometry and Epidemiology, Ludwig-Maximilians-University (LMU), Marchionini Str. 17, 81377 Munich Germany

Hölzel Dieter, Prof. Dr.

Munich Cancer Registry (MCR), Institute for Medical Information Processing, Biometry and Epidemiology, Ludwig-Maximilians-University (LMU), Marchionini Str. 17, 81377 Munich Germany

Engel Jutta, Prof. Dr.

Munich Cancer Registry (MCR), Institute for Medical Information Processing, Biometry and Epidemiology, Ludwig-Maximilians-University (LMU), Marchionini Str. 17, 81377 Munich Germany

Corresponding author: Dr. Kathrin Halfter, Munich Cancer Registry (MCR), Institute for Medical Information Processing, Biometry and Epidemiology, Ludwig-Maximilians-University (LMU), Marchionini Str. 17, 81377 Munich Germany. Email: halfter@ibe.med.uni-muenchen.de, Phone: +49 (0) 89 4400-74486, Fax: +49 (0) 89 4400-74753.

Supplementary Material

Table 1: Overview of the person yrs at risk and age-adjusted outcome parameters according to sex for the incidental CRC cases within the study cohort and the comparison population from the MCR.

	Total (n=10 889)						
	Person yrs at risk$_{Study}$	ASR$_{Screening}$	95% CI	ASR$_{MCR}$	95% CI	SIR	95%CI
**All ages**							
Male	65 573	24.1	15.3 – 33.0	73.4	72.2 – 74.6	0.33	0.27 – 0.40
Female	85 141	14.8	10.1 – 19.6	45.2	44.3 – 46.0	0.33	0.28 – 0.39
		TASR$_{Screening}$	95% CI	TASR$_{MCR}$	95% CI	TSIR	95%CI
**Age > 50 yrs**							
Male	61 373	69.0	43.3 – 94.7	208.4	204.9 – 211.8	0.33	0.27 – 0.40
Female	79 394	43.4	29.4 – 57.5	124.1	121.8 – 126.5	0.35	0.29 – 0.42

ASR, Age-standardized-rate, MCR, Munich Cancer Registry, SIR, Standardized incidence ratio, TASR, Truncated Age-Standardized-rate, TSIR, Truncated standardized incidence ratio.

Table 2: List of participating gastroenterologists

Name	Institution	Address
Amon, Karl	Gemeinschaftspraxis Dres.Seibold,Morell,Kurz,Kastner	Bahnhofstraße 10 82256 Fürstenfeldbruck
Andratschke, Klaus	Dres. K.Andratschke, S.Holle-Wölfel	Tegernseer Landstr. 11 82054, Sauerlach
Bauer, Guido	GZK Gesundheitszentrum Karlsfeld	Münchner Strasse 187 85757 Karlsfeld
Baumgartner, Reinhard	MVZ Kliniken Mühldorf	Krankenhausstr. 1a 84453, Mühldorf
Beyer, Tomas	Dr.med. T.Beyer	Reifenstuelstr. 4 83022, Rosenheim
Beyer, Albert	Praxis Dr. med. Albert Beyer	Mühldorfer Str. 14 84503 Altötting
Birkner, Berndt	Dr. Berndt Birkner	Einsteinstrasse 1 81675, München
Coenen, Christoph	MVZ St.Cosmas	Rathausplatz 1 85579 Neubiberg
Coutier, Wolfgang	Gemeinschaftspraxis Dr. med. W. Couturier und Dr. med. R. Ebeling	Hauptstraße 24 82256 Fürstenfeldbruck
Dettmer, Axel	Gastroenterologische Facharztpraxis am Rosenheimer Platz	Rosenheimer Platz 52 81669 München
Dichtl, Karl	Dr.med. K.Dichtl	Landshuter Straße 9 85435 Erding
Eimiller, Albert	Vorsorgezentrum Dr. Albert Eimiller Im Isar-Medizin-Zentrum	Sonnenstr. 24-26 80331 München
Fabian-Krause, Josef	Dr. med. J. Fabian-Krause	Prof.-Max-Lange-Platz 16 83646 Bad Tölz
Fach, Aldo	Dres. A.Fach, S.Reichel	Max-Josefs-Platz 21 83022 Rosenheim
Fischer, Burkhard	Dr. med. Burkhard Fischer	Hartstraße 52 82110 Germering

Folwaczny, C.	Dres.C.Folwaczny,<br>T.Sterzl,V.Brumberger	Isenschmidstr. 19<br>81545 München
Frankenberger, Ulrich	Dr.med. U.Frankenberger	Theodolindenstr. 97<br>81545 München
Götz, Hans	Dr.med. H.Götz	Töginger Straße 12<br>84453 Mühldorf
Groß, Felix	Dr.med. F.Groß	Reschstraße 2<br>82418 Murnau
Guth, Norbert	Dres. N.Guth, C.Huber	Kirchenstraße 7<br>82194 Gröbenzell
Hagena, Dirk	Innere Medizin Gilching	Pollinger Str. 23<br>82205 Gilching
Hobelsberger, Annette	Dr.med. A.Hobelsberger	Achdorfer Weg 5<br>84036 Landshut
Höchter, Wilhelm	Dres. C.Höchter, W.Höchter,<br>M. Schnürr	Landshuter Allee 43<br>80637 München
Hornberger, Joachim	Dr. J. Hornberger	Wittelsbacherstr. 10<br>83435 Bad Reichenhall
Huber, Frank	Dr.med. F.Huber	Kaspar-Aiblinger-Platz 36<br>83512 Wasserburg
Kammermeier, Rudolf	Internistische Gemeinschaftsprax.<br>an d. Klinik Dr. Schindlb.	Seestrasse 43<br>82211 Herrsching
Kittstein, Hermann	Dres. E.Glaser, M.Heindl,<br>H.Kittstein	Ludwigstraße 50<br>82467 Garmisch-Partenkirchen
Krumpoch, Bernd	Dres. B.Krumpoch, F.Trumm	Plinganserstr. 47<br>81369 München
Kurjak, Manfred	PD Dr.med. M.Kurjak	Friedenspromenade 40<br>81827 München
Landry, Wilfred	MVZ Dachau	Münchner Straße 64<br>85221 Dachau
Mergenthaler, Thomas	Dres. E.Mayer, T.Mergenthaler	Robert-Koch-Straße 54<br>84489 Burghausen
Munte, Axel	Ärztehaus Harlaching	Isenschmidstraße 19<br>81545 München
Ponton, Werner	Proktologie Am Marienplatz	Marienplatz 1<br>80331 München
Reichl-Glaß, Elisabeth	Dres. U.Bauer, E.Reichl-Glaß,<br>J.Stumpf	Untere Bahnhofstraße 53<br>82110 Germering
Reiter, Richard	Dr.med. R.Reiter	Fraunhoferstr. 6<br>80469 München
Resch, Werner	Dres. W.Resch, R.Richter	Ländgasse 137 – 138<br>84028 Landshut
Römer, Michael	Dr.med. M.Römer	Sendlinger Str. 21<br>80331 München
Ruckdeschel, Peter	Dres. K.Göttsberger,<br>P.Ruckdeschel	Karlsplatz 10<br>80335 München
Schatke, Winfried	Gemeinschaftspraxis<br>Roland Ott	Englschalkinger Str. 14<br>81925 München
Schögel, Edgar	Dres. R.Harsdorf Von<br>Enderndorf, E.Schögel	Sulzer Straße 7<br>82380 Peißenberg
Straka, Gernot	Gastroenterologie Ebersberg	Paul-Huber-Weg 2<br>85560 Ebersberg
Thelen, Hans-Arno	Medicen.Germ.S.Koemm,<br>B.Lautenschütz, C.Schiessel, H.Thelen	Hartstr. 52<br>82110 Germering
Weber-Guskar, Wolfgang	Gastroenterologie Starnbergersee	Bahnhofstraße 7

		82327
Wegerle, Wolfgang	Dres. A.Hainzinger,<br>E.Moroder,H.Schöppl, W.Wegerle	Räterstr. 20<br>85551 Kirchheim
Wenk, Andreas	Dres. M.Ibarra, H.Tittus,<br>B.Wachsmann, A.Wenk	Planegger Str. 4<br>81241 München
Wenner, Andreas	Dres. G.Mehltretter, A.Wenner	Thalbacher Straße 9 a<br>85368 Moosburg
Wolfer, Stefan	Dr.med. S.Wolfer	Karl-Theodor-Str. 95<br>80796 München
